# Effect of massive cerebellar infarction on the outcomes of patients with acute basilar artery occlusion during hospitalization after endovascular treatment: A retrospective study

**DOI:** 10.1097/MD.0000000000034154

**Published:** 2023-07-21

**Authors:** Chuyue Wu, Jing Wang, Lina Zhang, Fei Yan, Zhenjie Yang, Lei He, Jing Guo

**Affiliations:** a Department of Neurology, Chongqing University Three Gorges Hospital, Chongqing, P.R. China; b School of Medicine, Chongqing University, Chongqing, P.R. China; c Chongqing Municipality Clinical Research Center for Geriatric diseases, Chongqing University Three Gorges Hospital, Chongqing, P.R. China; d NHC Key Laboratory of Diagnosis and Treatment on Brain Functional Diseases, The First Affiliated Hospital of Chongqing Medical University, Chongqing, P.R. China; e Affiliated Yongchuan Hospital of Chongqing Medical University, Chongqing, P.R. China; f Department of Radiology, Chongqing University Three Gorges Hospital, Chongqing, P.R. China.

**Keywords:** acute basilar artery occlusion, endovascular treatment, massive cerebellar infarction, outcome

## Abstract

Acute basilar artery occlusion (ABAO) after endovascular treatment (EVT) is often associated with a poor prognosis, particularly in patients with cerebellar infarction who may develop malignant cerebellar edema. The present study aimed to investigate how massive cerebellar infarction (MCI) affects hospitalization outcomes in ABVO patients who undergo EVT. We conducted a retrospective study of ABVO patients who underwent EVT at our hospital between September 2017 and September 2022. MCI was diagnosed using imaging techniques, and various prognostic scores were assessed during hospitalization to examine the relationship between MCI and these outcomes. We identified 42 ABAO patients, of whom 22 (52.4%) had MCI. Patients with MCI had a higher modified Rankin Scale (mRS) score at discharge compared to those without MCI (4.36 ± 1.14 vs 3.05 ± 1.85, *P* = .042, odds ratio [OR] (95% confidence interval [CI]) = 1.093 (0.083, 2.103)), and a lower Glasgow Coma Scale score (6.59 ± 4.0 vs 10.10 ± 5.07, *P* = .036, OR (95% CI) = −3.444 (−6.518, −0.369)). MCI was identified as an independent risk factor for an extremely poor prognosis (mRS ≥ 5) at discharge (*P* = .036, OR (95% CI) = 15.531 (1.603, 313.026)) and for no improvement in mRS score compared to onset (*P* = .013, OR (95% CI) = 0.025 (0.001, 0.274)). Additionally, an extremely poor prognosis was independently associated with stent implantation, EVT duration, and body mass index, while mRS score improvement was correlated with EVT duration and pulmonary infection. MCI in ABAO patients is a significant independent risk factor for a poor prognosis at discharge and no improvement in function score compared to onset. Early diagnosis and intervention are necessary to improve outcomes, particularly in high-risk populations.

## 1. Introduction

Acute basilar artery occlusion (ABAO) is a type of acute ischemic stroke caused by atherosclerosis, thrombosis, embolus shedding or other factors, leading to acute ischemia of vital parts of the cerebellum, brain stem, thalamus, and other neurological deficits, with high disability and mortality rates.^[1,2]^ Studies have demonstrated that untreated ABAO patients have a fatality rate as high as 80% to 90%.^[3,4]^

Drugs are typically ineffective in treating ABAO, and the prognosis of patients undergoing endovascular treatment (EVT) is uncertain due to the high risk and technical difficulty involved. Both the endovascular treatment versus standard medical treatment for vertebro-basilar artery occlusion trial and the Basilar artery International Cooperation Study failed to show superiority of EVT over drug therapy in patients with posterior circulatory aorta occlusion.^[5,6]^ The EVT for Acute Basilar Artery Occlusion Study found EVT associated with better clinical outcomes in patients.^[7]^ EVT is a technically demanding procedure that necessitates the use of specialized equipment and expertise.^[8]^ Endovascular therapy improved functional outcomes in patients with large cerebral infarctions compared to medical care alone, but resulted in more intracranial hemorrhages.^[9]^ Optimizing EVT techniques to improve outcomes and reduce the risk of complications, for example, by developing new devices or approaches better suited to treating ABAO,^[9]^ could be the focus of research. Currently, researchers worldwide tend to treat stroke with posterior circulatory large vessel occlusion with EVT.

Although some patients’ short-term outcomes have improved thanks to EVT, nothing is known about how this surgery would affect patients’ quality of life and level of handicap over the long term.^[10,11]^ The usefulness of EVT in preventing impairment and enhancing quality of life in ABAO patients over the long term should be evaluated by additional research. Several factors, including age, stroke severity, time from reperfusion to onset, infarct volume before treatment, thrombosis length, etiology, and collateral circulation, may affect the clinical prognosis of ABAO patients after EVT.^[2,12–20]^ Therefore, the clinical prognosis may be comprehensively influenced by various factors.

In clinical practice, cerebellar infarction is frequently observed in ABAO patients, and such patients may develop malignant cerebellar edema (MCE), resulting in obstructive hydrocephalus and secondary brain stem damage due to the mass effect.^[21]^ Cerebellar infarctions in ABAO may progress to malignant cerebellar edoema MCE, resulting in obstructive hydrocephalus and a secondary injury to the brainstem due to mass effects.^[22]^ The mortality rate of MCE patients with acute cerebellar infarction is over 3 times higher than that of non-MCE patients according to a previous report.^[23]^ However, these studies focused on anterior circulation stroke, and there is limited evidence on ABAO patients. This study aimed to investigate the impact of massive cerebellar infarction (MCI) on hospitalization outcomes in ABVO patients after EVT at our center.

## 2. Methods

### 2.1. Study design

At the Neurological Department of Chongqing University Three Gorges Hospital, we conducted this retrospective analysis of patients who were prospectively enrolled in an ongoing cohort. The Chongqing University Three Gorges Hospital’s Clinical Trial Ethics Committee examined and approved the study protocols. All subjects or their substitutes gave their informed consent in accordance with the Helsinki Declaration. The study was registered and archived in the National Medical Research Registration and Archival Information System, with a unique identifier of MR-50-22-001641.

### 2.2. Participant recruitment

**Figure 1.**
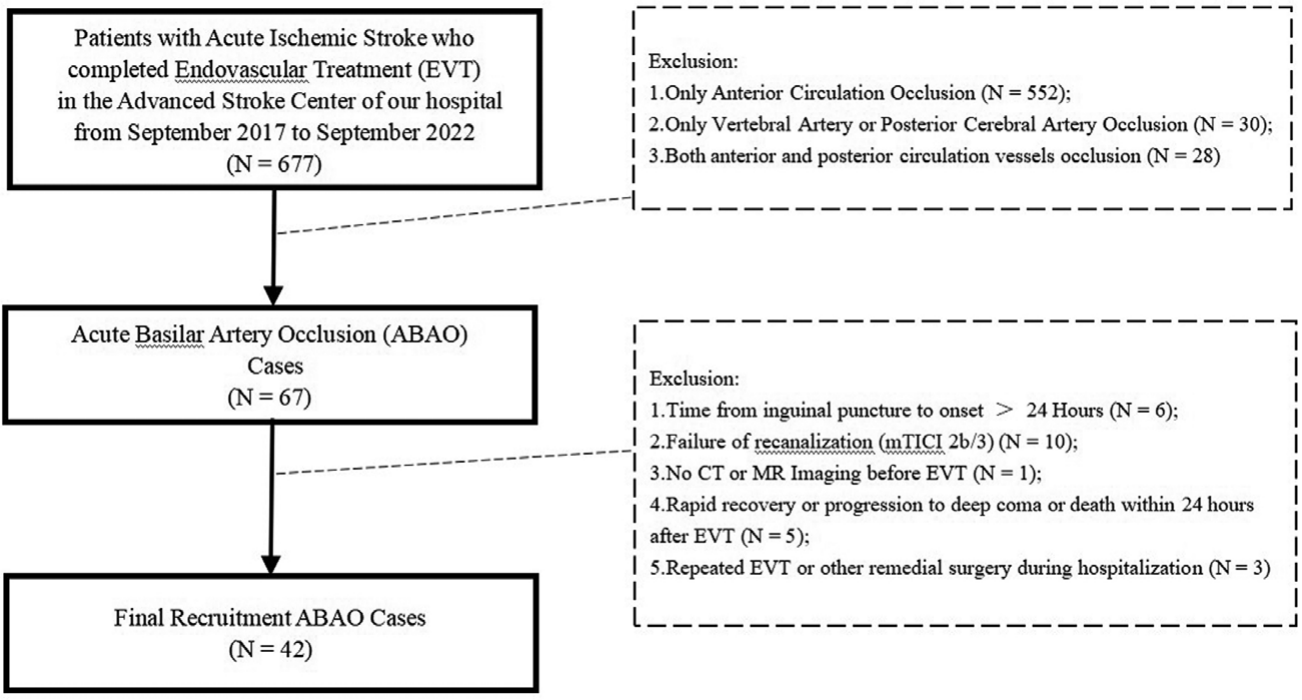
Flow diagram of eligibility of anterior circulation vessel occlusion cases.

The study included patients with ABAO who received EVT within 24 hours of symptom onset at the Advanced Stroke Center of our hospital between September 2017 and September 2022. Patients may have received intravenous thrombolysis or not. The flow chart of patient inclusion and exclusion was in Figure 1.

#### 2.2.1. Inclusion criteria.

ABAO confirmed by digital subtraction angiography; the time from inguinal puncture to onset was within 24 hours; recanalization occurred after EVT (mTICI grade 2b/3); complete CT or MR imaging examination before EVT; and clinical defects related to physician evaluation (without any score on scale limitation).

#### 2.2.2. Exclusion criteria.

Rapid recovery or progression to deep coma or death within 24 hours after EVT; and repeated EVT or other remedial surgery during hospitalization.

### 2.3. Clinical data

In this retrospective study, we collected data on various demographic factors, such as sex, age, body mass index (BMI), past medical history (including diabetes, hypertension, coronary heart disease, atrial fibrillation, hyperlipidemia, smoking, alcohol consumption, previous stroke, anticoagulant drug use, and antiplatelet drug use), duration of EVT, time from inguinal puncture to onset, length of hospital stay, and complications such as pulmonary infection and deep venous thrombosis. We also collected scale score data including the posterior circulation Acute Stroke Prognosis Early Computed Tomography Score,^[24]^ modified Rankin Scale (mRS) score^[25]^ (before onset, after onset, and at discharge), Glasgow Coma Scale (GCS) score^[26]^ (at admission, before EVT, after EVT, and at discharge), and National Institute of Health Stroke Scale (NIHSS) score^[27]^ (at admission, before EVT, after EVT, and at discharge).

We categorized hospitalization outcomes based on discharge scores: favorable prognosis (mRS ≤ 2), very unfavorable prognosis (mRS ≥ 5), and improvement in mRS (mRS at discharge < mRS at onset).^[25]^ We also evaluated GCS improvement, which was divided into 3 categories: GCS improvement 1 (GCS at discharge > GCS at admission), GCS improvement 2 (GCS at discharge > GCS before EVT), and GCS improvement 3 (GCS at discharge > GCS after EVT).^[26]^ Additionally, we assessed NIHSS improvement, which was classified into 2 categories: NIHSS improvement 1 (NIHSS at discharge < NIHSS at admission), NIHSS improvement 2 (NIHSS at discharge < NIHSS before EVT), and NIHSS improvement 3 (NIHSS at discharge < NIHSS after EVT).^[27]^

### 2.4. Imaging evaluation

**Figure 2.**
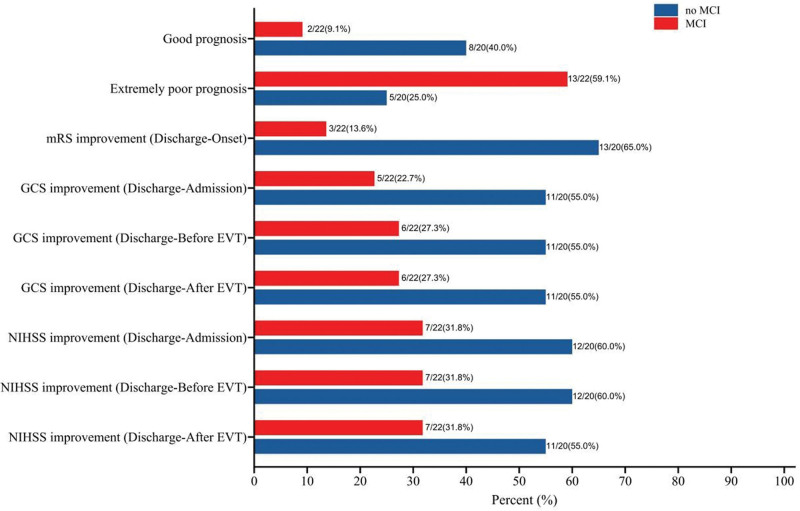
ROC curves and PR curves and overall quality of the 4 models. Model A: Only demographic and clinical data were included as predictors. Model B: On the basis of model A, serum biochemical indexes before EVT were included. Model C: On the basis of model A, serum biochemical indexes after EVT were included. Model D: Based on model A, serum biochemical indexes both before and after EVT were included. The AUC value of model D is the highest, and the PR curve shows good overall accuracy. The PR curve shows the least accurate model C, which is located in the middle of the graph. A good model has a value above 0.5. A value less than 0.5 indicates the model is no better than random prediction. Use caution in interpreting this chart since it only reflects a general measure of overall model quality. The model quality can be considered “good” even if the correct prediction rate for positive responses does not meet the specified minimum probability. Use the classification table to examine correct prediction rates. EVT = endovascular treatment, PR = precision-recall, ROC = receiver operating characteristic.

Two expert neuroradiologists were enlisted to assess CT or MR imaging conducted prior to EVT, specifically to identify the existence of MCI. The criteria for MCI diagnosis were that the infarct volume had to exceed 50% of the cerebellar volume, and evaluations were made at the level of cerebellar structures. The neuroradiologists classified the infarction as cerebellar or non-cerebellar, as shown in Figure 2. In the event of discrepancies between the 2 evaluators, a third neuroradiologist was called upon to confirm the findings. All neuroradiologists were blinded to the clinical outcomes while analyzing the imaging data.

### 2.5. Statistical analysis

The χ^2^ test was used to examine categorical variables. The Kolmogorov–Smirnov test was used to assess if continuous variables had a normal distribution. The Student’ *t* test was used to evaluate independent samples, while the Mann–Whitney *U* test was used to analyze non-normal data. The information was displayed as median with interquartile range, mean ± standard deviation, or number (percentage). An adjusted analysis was conducted, including age, sex, BMI, length of surgery, length of hospital stay, and past medical history, to examine the relationship between MCI and clinical outcome. Binary and multivariate logistic regression analyses were conducted to assess other independent risk factors for clinical outcome. The statistical tool used for these calculations was SPSS 22.0, with a significance level of *P* < .05.

## 3. Results

A total of 42 patients with ABAO were included in the study, of which 22 (52.4%) were diagnosed with MCI. Among all patients, 10 (23.8%) had a good prognosis, while 18 (42.9%) had an extremely poor prognosis at the time of discharge. The overall mRS improvement rate was 38.1%. The improvement rates of GCS at discharge were 38.1%, 40.5%, and 40.5% compared with admission, before EVT, and after EVT, respectively. The improvement rates of NIHSS score at discharge were 45.2%, 45.2%, and 42.9%, respectively, as mentioned previously.

Table 1 compares the hospital scores and other baseline data for patients with and without MCI. The results showed that the mRS score at discharge was significantly higher in patients with MCI compared to those without MCI (4.36 ± 1.14 vs 3.05 ± 1.85, *P* = .008), while the GCS score was significantly lower (6.59 ± 4.0 vs 10.10 ± 5.07, *P* = .017). In addition, there was a significant difference between patients with and without MCI in terms of smoking history (15 (68.20%) vs 4 (20.00%), *P* = .005) and alcohol consumption (11 (50.00%) vs 3 (15.00%), *P* = .038). Other data did not show significant differences between the 2 groups.

**Table 1:** Demographic data of patients with and without cerebellar infarction.

		No MCI (N = 20)	MCI (N = 22)	*P*
mRS after stroke		4.30 ± 0.66	4.05 ± 0.79	.260
mRS at discharge		3.05 ± 1.85	4.36 ± 1.14	.008*
Admission GCS		6.25 ± 3.52	6.36 ± 3.27	.915
Preoperation GCS		6.35 ± 3.57	6.23 ± 2.83	.903
Postoperation GCS		7.90 ± 3.64	6.27 ± 3.25	.136
Discharge GCS		10.10 ± 5.07	6.59 ± 4.07	.017*
Admission NIHSS		24.00 ± 8.90	21.32 ± 9.09	.340
Preoperation NIHSS		23.65 ± 8.53	21.50 ± 7.99	.406
Postoperation NIHSS		18.85 ± 9.98	21.95 ± 9.82	.317
Discharge NIHSS		14.40 ± 13.01	21.59 ± 11.87	.070
PC-ASPECT		6.90 ± 1.97	6.91 ± 1.54	.987
Duration of EVT, min		104.45 ± 39.57	132.64 ± 51.23	.052
Time from inguinal puncture to onset (h)		7.76 ± 6.39	8.60 ± 4.92	.289
Length of hospital stay (d)		16.05 ± 12.51	13.55 ± 14.96	.231
Age, yr		69.20 ± 13.81	65.82 ± 10.93	.388
BMI		22.32 ± 2.89	23.88 ± 3.14	.101
Stent implantation	0	14 (70.00%)	11 (50.00%)	.315
	1	6 (30.00%)	11 (50.00%)	
Balloon dilatation	0	13 (65.00%)	12 (54.55%)	.708
	1	7 (35.00%)	10 (45.45%)	
Intravenous thrombolysis	0	17 (85.00%)	19 (86.36%)	1.000
	1	3 (15.00%)	3 (13.64%)	
Gender	Male	14 (70.00%)	19 (86.40%)	.213
	Female	6 (30.00%)	3 (13.60%)	
Past history				
Smoking	0	16 (80.00%)	7 (31.80%)	.005*
	1	4 (20.00%)	15 (68.20%)	
Alcohol consumption	0	17 (85.00%)	11 (50.00%)	.038*
	1	3 (15.00%)	11 (50.00%)	
Hypertension	0	5 (25.00%)	10 (45.45%)	.289
	1	15 (75.00%)	12 (54.55%)	
Diabetes	0	14 (70.00%)	20 (90.91%)	.123
	1	6 (30.00%)	2 (9.09%)	
Coronary heart disease	0	14 (70.00%)	16 (72.73%)	1.000
	1	6 (30.00%)	6 (27.27%)	
Atrial fibrillation	0	15 (75.00%)	20 (90.91%)	.229
	1	5 (25.00%)	2 (9.09%)	
Hyperlipemia	0	15 (75.00%)	17 (77.27%)	1.000
	1	5 (25.00%)	5 (22.73%)	
Previous stroke	0	18 (90.00%)	22 (100.00%)	.221
	1	2 (10.00%)	0 (0.00%)	
Anticoagulant drug use	0	18 (90.00%)	22 (100.00%)	.221
	1	2 (10.00%)	0 (0.00%)	
Antiplatelet drug use	0	17 (85.00%)	22 (100.00%)	.099
	1	3 (15.00%)	0 (0.00%)	
Pulmonary infection	0	4 (20.00%)	2 (9.09%)	.400
	1	16 (80.00%)	20 (90.91%)	
Deep venous thrombosis	0	18 (90.00%)	18 (81.82%)	.665
	1	2 (10.00%)	4 (18.18%)	

BMI = body mass index, GCS = Glasgow Coma Scale, MCI = massive cerebellar infarction, mRS = modified Rankin Scale, NIHSS = National Institute of Health Stroke Scale, pc-ASPECTS score = posterior circulation Alberta Stroke Program Early CT Score.

*P < 0.05.

The results of the comparison between patients with and without MCI regarding clinical outcomes are presented in Figure 2. The data revealed significant differences in terms of the rates of good prognosis (9.1% vs 40.0%, *P* = .029), extremely poor prognosis (59.1% vs 25.0%, *P* = .030), mRS improvement (13.6% vs 65.0%, *P* = .002), and GCS improvement 1 (22.7% vs 55.0%, *P* = .036). The rates of improvement in GCS 2 (27.3% vs 55.0%, *P* = .072), GCS 3 (27.3% vs 55.0%, *P* = .072), NIHSS score 1 (31.8% vs 60.0%, *P* = .071), NIHSS score 2 (31.8% vs 60.0%, *P* = .071), and NIHSS score 3 (31.8% vs 55.0%, *P* = .133) were lower in patients with MCI than those without MCI, but the differences were not statistically significant.

**Figure 3.**
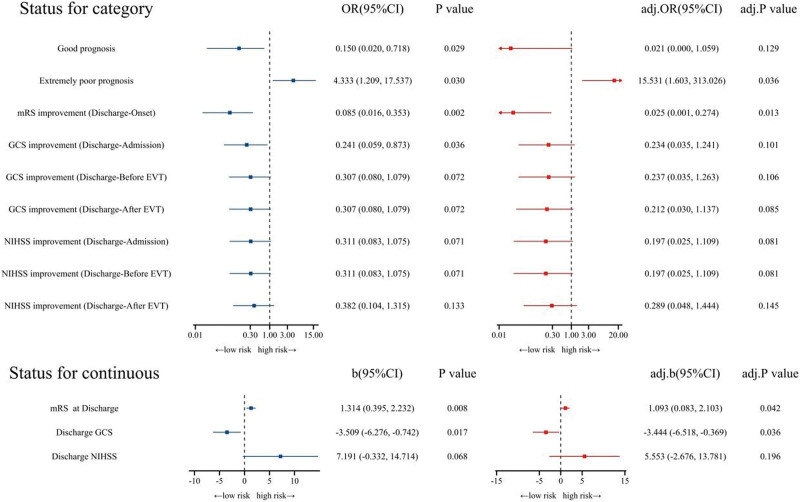
Forest map-adjusted analysis of massive cerebellar infarction predicting discharge outcome. Good prognosis (mRS at discharge ≤ 2), extremely poor prognosis (mRS at discharge ≥ 5). For example, GCS improvement (Discharge-Admission) means GCS at discharge > GCS at admission, while NIHSS improvement (Discharge-Admission) means NIHSS at discharge < NIHSS at admission. The rest of the definitions are similar. Adjusted analysis including age, sex, BMI, length of surgery, length of hospital stay, and past medical history. BMI = body mass index, CI = confidence interval, EVT = endovascular treatment, GCS = Glasgow Coma Scale, mRS = modified Rankin Scale, NIHSS = National Institute of Health stroke scale, PC-ASEPTS = posterior circulation Acute Stroke Prognosis Early Computed Tomography Score.

MCI was found to be an independent risk factor for multiple clinical outcomes after adjusted analysis. Specifically, patients with MCI had a higher mRS score at discharge (adj. *P* = .042, adj. odds ratio [OR] (95% confidence interval [CI]) = 1.093 (0.083, 2.103)) and a lower GCS score at discharge (adj. *P* = .036, adj. OR (95% CI) = −3.444 (−6.518, −0.369)). They were also more likely to have an extremely poor outcome at discharge (adj. *P* = .036, adj. OR (95% CI) = 15.531 (1.603, 313.026)) and less likely to show improvement in mRS (adj. *P* = .013, adj. OR (95% CI) = 0.025 (0.001, 0.274)). These results are presented in Figure 3.

Table 2 presents the logistic regression analysis results for the association between other clinical factors and extremely poor prognosis at discharge and mRS improvement. The analysis revealed that stent implantation (*P* = .041, OR (95% CI) = 4.714 (1.255, 17.709)), EVT duration (*P* = .041, OR (95% CI) = 1.024 (1.002, 1.048)), and BMI (*P* = .024, OR (95% CI) = 1.289 (1.024, 1.624)) were independently associated with an extremely poor prognosis at discharge. Furthermore, EVT duration (*P* = .014, OR (95% CI) = 0.980 (0.962, 0.999)) and pulmonary infection (*P* = .023, OR (95% CI) = 0.088 (0.009, 0.844)) were independently correlated with mRS improvement.

**Table 2:** Logistic regression analysis of extremely poor prognosis and mRS improvement among other clinical factors influencing.

		Discharge mRS ≤ 4 (N = 24)	Discharge mRS ≥ 5 (N = 18)	*P*	OR (95% CI)
Stent implantation	0	18 (75.00%)	7 (38.89%)	.041	4.714 (1.255, 17.709)
	1	6 (25.00%)	11 (61.11%)		
Duration of EVT, min		105.71 ± 39.45	137.22 ± 52.67	.041	1.024 (1.002, 1.048)
BMI		22.21 ± 2.90	24.37 ± 2.98	.024	1.289 (1.024, 1.624)
		**No mRS improvement (N = 26**)	**mRS improvement (N = 16**)		
Duration of EVT, min		132.00 ± 50.97	98.44 ± 33.75	.014	0.980 (0.962, 0.999)
Pulmonary infection	0	1 (3.85%)	5 (31.25%)	.023	0.088 (0.009, 0.844)
	1	25 (96.15%)	11 (68.75%)		

BMI = body mass index, CI = confidence interval, EVT = endovascular treatment, mRS = modified Rankin Scale.

mRS improvement (mRS at discharge < mRS after onset).

**Figure 4.**
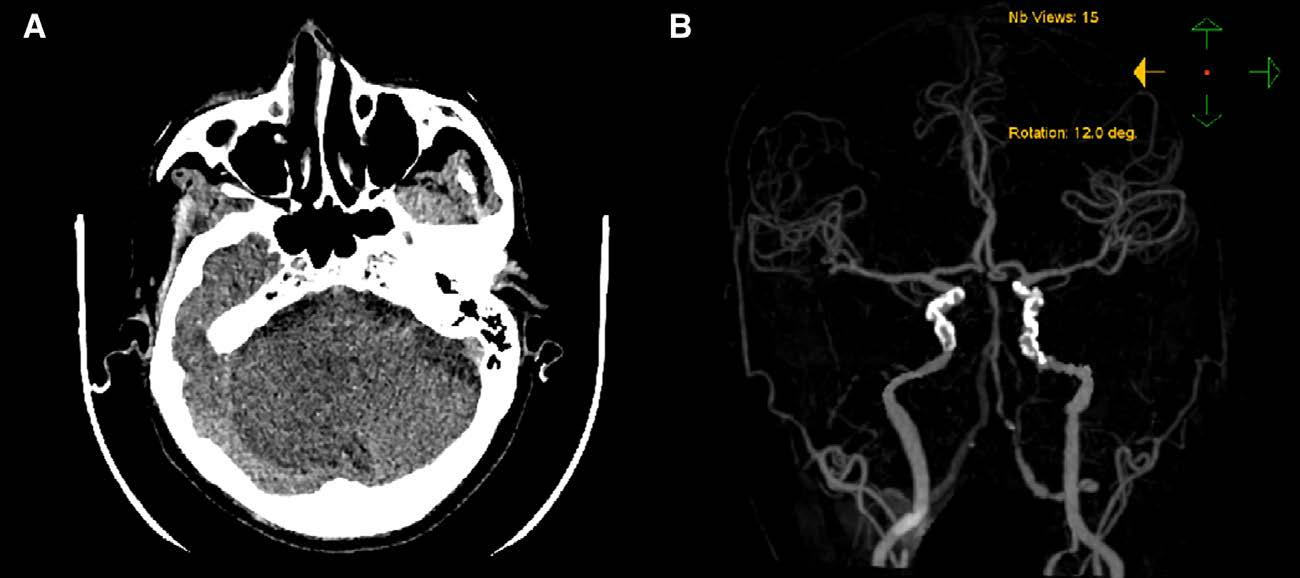
A 70-year-old elderly man with MCI including in our study, presented with a sudden disturbance of consciousness for 5 h. (A) Cranial CT suggested massive cerebellar infarction on the right side at admission. (B) Cranial CTA suggested basilar artery occlusion. The patient was conscious on the 1st day after EVT, but lost consciousness and entered a deep comatose state on the 2nd day. The family refused further treatment and was discharged soon thereafter. CT = computed tomography, CTA = computed tomography angiography, EVT = endovascular treatment, MCI = massive cerebellar infarction.

**Figure 5.**
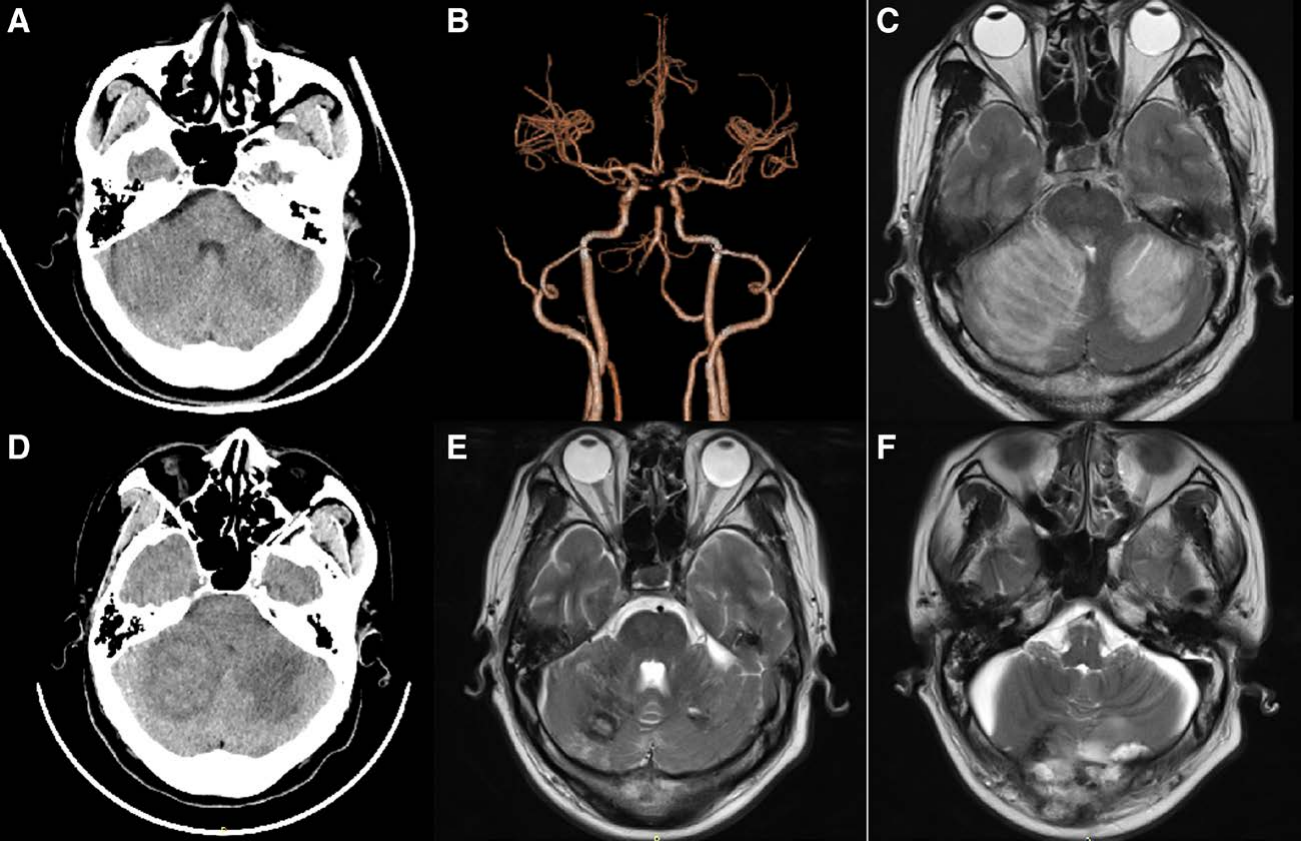
A 46-year-old man with MCI including in our study, presented with sudden dizziness and vomiting that lasted for 12 h and disturbance of consciousness that lasted for 2 h. (A) Cranial CT suggested bilateral massive cerebellar infarction at admission. (b) Cranial CTA suggested basilar artery occlusion. (C) After EVT, the patient was conscious, and cranial MRI still indicated bilateral massive cerebellar infarction. (D) On the 2nd night after EVT, loss of consciousness deepened, and cranial CT indicated cerebellar infarction with a space-occupying effect. The family consented to decompressive craniectomy. (E) At 3 weeks after craniectomy, the patient was conscious and could get out of bed and had a mRS score of 2 points. The cranial MRI indicated a bilateral cerebellar infarction that had been reduced. (F) The patient had a mRS score of 1 at discharge, and the cranial MRI indicated a further reduction in the area of bilateral cerebellar infarction. CT = computed tomography, CTA = computed tomography angiography, EVT = endovascular treatment, MCI = massive cerebellar infarction, MRI = magnetic resonance imaging, mRS = modified Rankin Scale.

Figures 4 and 5 present 2 cases from this study. Patient 1 (Fig. 4), the pre-EVT CT scan revealed a significant cerebellar infarction. Although the patient regained consciousness briefly after EVT, they soon experienced consciousness disturbance again. Despite the recommendation for a craniectomy, the patient’s family refused, and the patient passed away a few hours later. In contrast, Patient 2’s family (Fig. 5) consented to a decompressive craniectomy when the patient returned to a comatose state. At discharge, the patient had a mRS score of 1. Therefore, we recommend that for patients with ABAO, a head CT or MR should be reviewed early on both before and after EVT. Surgical intervention, combined with decompressive craniectomy, may improve the clinical prognosis for patients with massive cerebellar infarction. In our hospital, we plan to conduct follow-up studies to validate these observations.

## 4. Discussion

Our study emphasizes the significant impact of cerebellar infarction on the outcomes of patients with ABAO during hospitalization after EVT. Specifically, our results revealed that MCI was an independent risk factor for an extremely poor outcome at discharge and no improvement in function compared to admission.

The good prognosis rate in our study was 23.8%, while the extremely poor prognosis rate was 42.9%. Additionally, the overall mRS improvement rate was only 38.1%. In previous studies of ABAO patients who underwent EVT, prognosis varied considerably. For instance, a multicenter registry of 148 patients who underwent EVT reported that 79% achieved recanalization, and 34% achieved a good prognosis.^[12]^ Another multicenter registry study showed that 80% of patients achieved good vascular recanalization, with a 90-day good prognosis rate of 35%.^[14]^ This suggests that stent thrombectomy and aspiration thrombectomy could lead to good vascular recanalization.^[16,28]^ Mourand et al^[16]^ reported on 31 patients with ABAO who completed EVT with a Solitaire stent. The results showed that 74% of patients achieved vascular recanalization, and 35% of patients achieved a good prognosis. Yoon et al^[13]^ reported on 50 ABAO patients who underwent endovascular stent thrombus removal. Among them, 48 patients achieved vascular recanalization, with 27 patients having good prognoses and 6 patients dying.

Previous research has identified several factors that can affect the outcomes of EVT in ABAO patients. One important factor is the duration of treatment, with earlier initiation of EVT associated with better outcomes. For instance, a study by Vergouwen et al^[29]^ in 2012 found that the risk of poor prognosis increased with longer delays in achieving vascular recanalization, with EVT initiated more than 6 hours after onset being particularly associated with poor outcomes. In a multicenter retrospective study, Dorňak et al^[30]^ reported that longer treatment duration was independently associated with better prognosis at both 30 and 90 days. The functional result and death can be predicted by the NIHSS score at admission, the number of thrombectomy device passes, the postoperative pons-midbrain index, diabetes mellitus, and lung infection. Positive results were substantially correlated with both lung infection and NIHSS score upon admission. The NIHSS score at admission, the quantity of thrombectomy device passes, the postoperative pons-midbrain index, and diabetes mellitus were all linked to mortality.^[31]^ A positive result was significantly predicted by the admission NIHSS score (OR 0.98, 95% CI 0.97–0.099; *P* = .032). After thrombectomy, incomplete recanalization significantly increased the risk of dying (OR 1.68, 95% CI 1.18–2.39; *P* = .005).^[32]^ Ineffective recanalization was a reliable indicator of mortality. The mortality rate for patients who had successful recanalization was lower (32.9% vs 74.4%; *P* .001) (OR 5.1; 95% CI 1.34–19.33), and Lack of effective recanalization was a standalone predictor of mortality.^[33]^

Other studies have also identified baseline NIHSS score as a significant predictor of clinical prognosis in patients undergoing mechanical thrombectomy for acute posterior circulation ischemia. For example, in a multicenter registry study, patients with a median NIHSS score of 9 points had a good prognosis, while those with a median NIHSS score of 24 points had a poor prognosis.^[30]^ The NIHSS score was also found to be an independent predictor of good prognosis in ABAO patients, according to multivariate regression analysis. However, not all studies have reported consistent findings regarding the impact of treatment time on clinical prognosis in ABAO patients undergoing EVT. While some studies have found that earlier EVT initiation and shorter treatment times are associated with better outcomes,^[14,29,30]^ other studies have not found a significant association between treatment time and clinical prognosis.^[12,13,19]^

In a 2015 study, Yoon et al^[13]^ found that the NIHSS score was an independent predictor of clinical prognosis with an odds ratio of 0.82 and a *P* value of .008. Tei et al^[20]^ proposed the posterior circulation Alberta Stroke Program Early CT Score (pc-ASPECTS score) based on DWI in 2010 to evaluate its predictive value for clinical outcomes in patients with acute posterior circulation stroke. They concluded that both the PC-ASPECT score and NIHSS score were independent predictive factors for clinical outcomes. In our study, we did not find any correlation between the baseline NIHSS score or PC-ASPECT score and the clinical outcome, which may be attributed to the small sample size and the choice of outcome measure. However, we did find that poor prognosis at discharge was independently associated with the duration of EVT, BMI of the patient, and whether stenting was performed through EVT. Additionally, improvement in mRS score was independently correlated with the duration of EVT and the occurrence of pulmonary infection. Prolonged operative time may be due to multiple intraoperative thrombectomies or complicated special conditions and the use of remedial measures such as stenting, which may lead to a poor short-term prognosis. Obesity, as indicated by a high BMI, is a known risk factor for poor prognosis of cardiovascular disease.^[34]^ Patients with pulmonary infection often require symptomatic supportive treatment, leading to slow functional recovery.^[35]^

Few studies have examined the prognosis of ABAO patients with cerebellar infarction following EVT. The EVT for Acute Basilar Artery Occlusion study’s multicenter cohort served as the basis for Liu et al^[22]^ to be the first to investigate the relationship between MCE progression and clinical outcomes in ABAO cerebellar infarction patients. They concluded that MCE was an independent risk factor for functional deterioration in ABAO patients 90 days after EVT. Furthermore, Mourand et al^[23]^ found that cerebellar infarction on DWI prior to endovascular treatment was an independent predictor of 90-day mortality in ABAO patients, and an initial cerebellar infarction volume ≥ 4.7 mL was the optimal threshold for the risk of death. Our study previously presented additional evidence that the presence of cerebellar infarction was an independent predictor of poor or worsening functional outcomes in patients.

In this study, it was observed that the detection of massive cerebellar infarction before EVT was an indicator of poor outcomes during the acute phase of basilar artery occlusion thrombectomy. The reason behind this negative prognosis is believed to be the mass effect that occurs due to cerebellar infarction. Occupying cerebellar edema is one of the most severe and life-threatening complications of ABAO,^[21]^ and because of the limited space available in the posterior fossa, the cerebellar mass effect can directly compress the brainstem and fourth ventricle. This can lead to obstructive hydrocephalus, which can worsen the disease in the early stages and can occur alone or in combination with the aforementioned situations.

Even with active drug therapy, up to 48% of patients with cerebellar infarction displaying obvious space-occupying effects still experience clinical deterioration.^[36]^ The prognosis for massive cerebellar infarction can be improved through early decompressive craniectomy. A meta-study found that lower mortality rates and a reduced risk of adverse outcomes were associated with higher GCS scores, craniectomy performed within 48 hours of onset, age less than 60 years, and completion of external ventricular drainage to remove necrotic brain tissue.^[37]^ However, clinicians often exclude comatose patients or those with low neurological scores before the surgery due to their association with ABAO.

To improve outcomes for ABAO patients, we should enhance the diagnostic accuracy of imaging studies and identify abnormalities other than vascular occlusion as soon as possible. Early intervention strategies should be implemented for high-risk populations, in conjunction with evaluating other clinical manifestations and indicators, to improve hospitalization outcomes and potentially improve long-term prognosis for patients. ABAO frequently manifests with nonspecific signs and symptoms (e.g., vertigo, cephalalgia, decreased consciousness, or hemiparesis) that can mimic an anterior circulation stroke. To make an accurate diagnosis, imaging studies such as computed tomography, computed tomography angiography, and diffusion-weighted magnetic resonance imaging must be obtained as part of the diagnostic approach.^[38]^

Our study had some limitations that need to be considered. Firstly, it was a single-center, retrospective, observational study, which may limit the generalizability of our findings. Secondly, the small sample size made it difficult to determine if the results could be replicated in larger populations. Thirdly, we used functional scores at discharge as the primary outcome measure, but future studies should investigate the relationship between cerebellar infarction and long-term prognosis beyond 3 months. Finally, our definition of massive cerebellar infarction was based on a synthesis of several previous studies on cerebellar infarction,^[22,23,36,37]^ and further studies with larger sample sizes and controlled designs are necessary to confirm whether it is a reliable predictor of poor outcomes.

In conclusion, MCI in ABAO patients after EVT was an independent risk factor for poor prognosis at discharge and no improvement in function compared to post onset. Additionally, the stent implantation, duration of EVT, BMI, and the pulmonary infection were also found to be a significant factor in determining patient prognosis.

## Acknowledgments

We appreciate the imaging technicians for acquiring the high-quality images, the neurologists for assisting us, and all the participants for taking part in the study.

## Author contributions

**Conceptualization:** Chuyue Wu, Jing Guo.

**Data curation:** Chuyue Wu, Jing Wang, Lina Zhang, Fei Yan, Zhenjie Yang.

**Formal analysis:** Jing Wang, Lina Zhang, Lei He.

**Funding acquisition:** Jing Guo.

**Investigation:** Chuyue Wu, Jing Wang, Fei Yan, Zhenjie Yang, Lei He.

**Methodology:** Chuyue Wu, Jing Wang, Lina Zhang, Fei Yan, Lei He.

**Project administration:** Jing Wang, Fei Yan.

**Resources:** Lina Zhang, Jing Guo.

**Software:** Zhenjie Yang, Lei He, Jing Guo.

**Supervision:** Jing Guo.

**Validation:** Jing Wang, Lina Zhang, Zhenjie Yang.

**Visualization:** Fei Yan.

**Writing – original draft:** Chuyue Wu, Jing Wang.

**Writing – review & editing:** Chuyue Wu, Jing Wang, Lina Zhang, Fei Yan, Zhenjie Yang, Lei He, Jing Guo.
